# Azithromycin-Induced Liver Injury in Legionnaires' Disease

**DOI:** 10.7759/cureus.17856

**Published:** 2021-09-09

**Authors:** Krista M Wong, Keivan Hosseinnejad, Poornanand Palaparty, Keyvan Ravakhah

**Affiliations:** 1 Internal Medicine, St. Vincent Charity Medical Center, Cleveland, USA; 2 Hematology/Oncology, St. Vincent Charity Medical Center, Cleveland, USA

**Keywords:** drug-induced liver injury, legionella infection, legionnaires disease, community acquired pneumonia, azithromycin, ceftriaxone, antibiotics, hepatotoxicity, hepatocellular liver injury

## Abstract

Drug-induced liver injury (DILI) is the leading cause of acute liver failure in the United States. Azithromycin is a commonly used antibiotic for community-acquired pneumonia that causes liver injury in rare cases. Typically, cholestatic liver injury has been reported for azithromycin, but there have only been a few case reports addressing the association with direct hepatocellular liver injury. This is a case of a 66-year-old man, with no pre-existing liver disease, who was managed for Legionnaires’ disease who sustained a hepatocellular pattern of liver injury associated with azithromycin. We report this case to highlight the importance of prompt recognition of these rare side effects associated with azithromycin and the discontinuation of the drug to facilitate rapid recovery.

## Introduction

Drug-induced liver injury (DILI) is the most common cause of acute liver failure (ALF) in the United States [1]. It accounts for approximately 50% of ALF cases and 1-2% of hospital admissions [2,3]. Acetaminophen is the leading cause of DILI in the United States, followed by antibiotics most notably beta-lactams, macrolides, and sulfonamides [1,4]. Ceftriaxone and azithromycin are commonly used antibiotics for the empiric treatment of community-acquired pneumonia (CAP) to cover intracellular pathogens such as Legionella pneumophila. These antibiotics are associated with rare DILI and are most commonly cholestatic. There are only a few case reports on the direct hepatocellular injury associated with azithromycin [5,6]. The majority of patients experience complete recovery after prompt discontinuation of the offending drug.

Herein, we report the case of a man with no pre-existing liver disease who was treated with ceftriaxone and azithromycin for Legionnaires’ disease who sustained a hepatocellular pattern of liver injury with quick recovery after drug discontinuation. This case aimed to bring awareness to rare side effects associated with commonly used antibiotics that may have severe complications if not rapidly recognized and treated.

## Case presentation

A 66-year-old man with a past medical history of type 2 diabetes mellitus, hypertension, hyperlipidemia, and treated stage 4 follicular lymphoma (completed bendamustine and rituximab in 2018) presented with a three-day history of non-radiating, constant substernal chest tightness associated with fatigue, fever, and chills. He denied shortness of breath, coughs, abdominal pain, nausea, vomiting, diarrhea, myalgias, arthralgias, weight loss, appetite loss, genitourinary symptoms, or leg swelling. He had no known drug allergies and was compliant with his medications. His medications were moderate-intensity atorvastatin, amlodipine, lisinopril, metformin, and glipizide. He denied the use of acetaminophen, over-the-counter medications, or herbal medications. The patient also denied alcohol, tobacco, or illicit drug use. He had no known history of liver disease. Family history was noncontributory.

On admission, the patient had a fever of 39.4ºC, heart rate was 105/min, respiratory rate was 20/min, blood pressure was 130/74, and 95% oxygen saturation on room air with body mass index of 27. Physical examination was remarkable for decreased breath sounds and coarse, inspiratory crackles both present in the left lung base. The cardiovascular examination was unremarkable except for sinus tachycardia. Abdominal examination was soft and nontender with no hepatosplenomegaly. No peripheral stigmata of chronic liver disease. No lymphadenopathy was present.

Initial labs revealed a hepatocellular pattern of liver injury. Serum aspartate aminotransferase (AST) was 295 U/L (reference range: 15-37), alanine aminotransferase (ALT) was 236 U/L (reference range: 13-61), alkaline phosphatase (ALP) was 96 U/L (reference range: 45-117), and total bilirubin was 2.1 mg/dL (reference range: 0.2-1). Prothrombin time test (PT), international normalized ratio (INR), and albumin were within normal limits suggesting normal synthetic function of the liver. Complete blood count and electrolytes were within normal limits. Iron saturation was 13% (reference range: 25-35) and ferritin was elevated at 6102 ng/mL (reference range: 26-388).

EKG showed sinus tachycardia with no acute ST/T-wave changes. Troponins were negative. Chest x-ray showed left basilar airspace consolidation. Abdominal ultrasound showed mild hepatic steatosis with no evidence of gallstones or dilated bile ducts. The patient was started on empiric antibiotics, IV ceftriaxone (1 g every 24 hours) and IV azithromycin (500 mg every 24 hours), and intravenous fluids. Legionella urinary antigen was positive for Legionella pneumophila (subtype 1). Pneumococcal urinary antigen and blood cultures were negative. The initial hepatocellular liver injury was attributed to Legionella infection.

After receiving two doses of ceftriaxone and azithromycin, the patient’s liver transaminases suddenly spiked to AST 1135 U/L and ALT 1035 U/L with a mild elevation of ALP to 192 U/L. Total bilirubin continued to downtrend from admission to normal limits. Ferritin also concurrently increased to 34954 ng/mL. Clinically, the patient was asymptomatic and improved since admission. His presenting symptoms had resolved and he did not complain of gastrointestinal symptoms.

Antibiotics were discontinued and switched to oral levofloxacin (750 mg daily). Hepatotoxic medications were avoided and atorvastatin was held. The liver transaminases immediately downtrended the following day to AST 406 U/L and ALT 632 U/L. Ferritin decreased to 3802 ng/mL. ALP slightly downtrended during the admission approaching normal. 

Other differential diagnoses for acute hepatitis were ruled out. The patient's blood pressure was within normal limits during the admission and did not experience any drops that would precipitate ischemic hepatitis. Acetaminophen level was negative. Serum alcohol was negative. Urine toxicology was negative. Acute viral hepatitis was ruled out. IgM anti-hepatitis A virus was negative. Hepatitis B surface antigen (HBsAg) and IgM anti-hepatitis B core antigen (anti-HBc) were negative. Hepatitis C viral RNA and anti-hepatitis C virus antibody (HCV) were negative. The human immunodeficiency virus (HIV) fourth-generation test was negative. Epstein-Barr virus (EBV) IgM, cytomegalovirus (CMV) IgM, and varicella-zoster virus (VZV) IgM were negative. Herpes simplex virus types 1 and 2 (HSV 1/2) DNA polymerase chain reaction (PCR) were negative. Autoimmune hepatitis and Wilson disease were unlikely given the clinical picture and downtrend of liver transaminases after discontinuation of the inciting antibiotics. 

The patient was discharged on the eighth day of admission. During his four-week follow-up visit, he was shown to have a complete recovery. His liver transaminases and ALP were within normal limits.

## Discussion

When prescribing these commonly used antibiotics, it is important to be aware of rare side effects that may pose serious complications and to prescribe with caution for patients who may be at higher risk. We presented a patient with no history of liver disease who developed a hepatocellular pattern of liver injury that was likely drug-induced given the sudden spike of liver transaminases after administration of antibiotics, with quick resolution after their discontinuation. Furthermore, the Naranjo Probability Scale indicates a probable causal relationship between the drug and clinical event. Indeed, the drug’s side effect may have been idiopathic, but it is worth acknowledging the possibility of an underlying predisposition that may have rendered this patient more susceptible.

Our patient had slightly elevated liver transaminases and markedly elevated ferritin upon admission likely due to Legionella infection. Legionnaires’ disease is more frequently associated with elevated liver enzymes and ferritin as compared to non-Legionella CAP [7,8]. Perhaps this initial insult to the liver by Legionella infection predisposed this patient to drug-induced liver injury. Our patient had no pre-existing liver disease such as hepatitis B or C, HIV, or alcohol use disorder that might have made him more susceptible.

While it is known that ceftriaxone is a third-generation cephalosporin that is associated with gallbladder sludge and cholelithiasis in 3-46% of patients, most often seen in children, there have been some reports in adults [9]. Other risk factors include higher doses (>40 mg/kg/day or >2 g) and prolonged duration of treatment (>5 days). It can also cause an immunoallergic form of cholestatic hepatitis in very rare cases, which is usually mild and self-limited.

Given that our patient displayed a hepatocellular pattern rather than the cholestatic pattern of liver injury, the offending agent is more likely to have been azithromycin. Azithromycin is a semisynthetic macrolide that is associated with an asymptomatic elevation of liver transaminases in 1-2% of patients for those treated for short periods and a higher proportion for those with prolonged treatment [10]. Typically, azithromycin can cause cholestatic hepatitis, but it can also cause hepatocellular injury with jaundice [10,11]. The mechanism is not well-understood and is not dose-related. Hepatocellular injury is associated with a worse prognosis than that for pure cholestatic injury as it can be severe and lead to death or need for emergency liver transplantation [2]. Latency is typically short and can occur within days of drug exposure. Recovery in most cases occurs within four to eight weeks. Furthermore, azithromycin is predominantly hepatically eliminated. Although there are no dosage adjustment recommendations for hepatic impairment, one should exercise caution when prescribing due to the potential for hepatotoxicity.

## Conclusions

Antibiotics are commonly used medications that have rare side effects, as demonstrated in this report, which highlights the importance of vigilance when prescribing to patients who may be at higher risk. To date, there have only been a few case reports on the direct hepatocellular injury associated with azithromycin. Awareness of these side effects and how they present will enable healthcare professionals to promptly discontinue the offending drug to prevent severe, life-threatening complications.
